# Integrated Venom Gland Transcriptomic and Venom Proteomic Analyses of the Digger Wasp *Cerceris japonica*

**DOI:** 10.3390/toxins18070307

**Published:** 2026-07-15

**Authors:** Kohei Kazuma, Naoki Tani, Katsuhiro Konno, Migaku Kawaguchi, Hidetoshi Inagaki

**Affiliations:** 1 General Research Institute, Okinawa Churashima Foundation, 888 Aza Ishikawa, Motobu-cho, Kunigami-gun, Okinawa 905-0206, Okinawa, Japan; k-kazuma@okichura.jp; 2 Liaison Laboratory Research Promotion Center, Institute of Molecular Embryology and Genetics, Kumamoto University, 2-2-1 Honjo, Chuo-ku, Kumamoto 860-0811, Kumamoto, Japan; naotani@kumamoto-u.ac.jp; 3 Graduate School of Agricultural Science, Tohoku University, 468-1 Aramaki Aza Aoba, Aoba-ku, Sendai 980-8572, Miyagi, Japan; katsuhiro.konno.b8@tohoku.ac.jp; 4 Research Institute for Material and Chemical Measurement, National Metrology Institute of Japan (NMIJ), National Institute of Advanced Science and Technology (AIST), 1-1-1 Umezono, Tsukuba 305-8563, Ibaraki, Japan; m-kawaguchi@aist.go.jp; 5 Molecular Biosystems Research Institute, National Institute of Advanced Science and Technology (AIST), 1-1-1 Higashi, Tsukuba 305-8566, Ibaraki, Japan

**Keywords:** digger wasp, apoid wasp, *Cerceris japonica*, hymenoptera, venom evolution

## Abstract

Digger wasps are solitary apoid wasps that excavate ground nests and use venom to paralyze insect prey. Recent phylogenetic analyses suggest that digger wasps belong to an ancient lineage of aculeate Hymenoptera and share an evolutionary ancestry with other venomous hymenopterans, including bees and ants. Although the venoms of ants, bees, and social wasps have been extensively studied, those of digger wasps remain poorly characterized in terms of their composition, molecular diversity, and biological activity. In this study, we investigated the venom of the digger wasp *Cerceris japonica* using integrated transcriptomic and proteomic analyses. We identified 19 toxin-like proteins and peptides, 14 non-toxin-associated components, and 11 novel peptides and proteins with no detectable similarity to known peptides and proteins. Among these, peptide *Cj* 2 exhibited insecticidal activity but showed no antimicrobial, hemolytic, or nicotinic acetylcholine receptor-modulating activities. These findings provide new insights into the molecular diversity and biological activities of digger wasp venom, expand our understanding of venom evolution in venomous hymenopterans, and serve as a framework for elucidating the conserved and lineage-specific features of hymenopteran venoms.

## 1. Introduction

Digger wasps (apoid wasps) are predatory solitary hymenopterans that use venom to paralyze their prey for larval provisioning. Although the venoms of ants, bees, and social wasps have been extensively studied, relatively little is known about the composition, molecular diversity, and biological activity of digger wasp venoms. Several bioactive components have been identified in digger wasps, including philanthotoxins from *Philanthus triangulum*, FMRFamide-related neuropeptides from *Sphex argentatus* and *Isodontia harmandi* [1]. In addition, transcriptomic and proteomic analyses have revealed numerous toxin-like peptides and proteins in other digger wasp species [2,3]. However, the venom composition of most digger wasp species is largely unknown.

The digger wasp species *Cerceris japonica* (Crabronidae) [4] is an endemic Japanese species whose larvae primarily feed on sweat bee larvae (Halictidae). In previous studies, we characterized the venom components of ants [5,6] and solitary wasps [1]. As ants and digger wasps are phylogenetically closely related and share unique behaviors, such as nesting underground and preying on insects to provision their larvae, we hypothesized that comparing their venom components could provide insights into these behaviors. Furthermore, comparative studies of venomous hymenopterans, including ants, bees, and wasps, have provided important insights into venom evolution. Considering their shared evolutionary ancestry within Aculeata and partially convergent ecological traits, a comparison of venom components between ants and digger wasps may help clarify the conserved and lineage-specific features of hymenopteran venoms.

In this study, we characterized the venom composition of *C. japonica* using integrated transcriptomic and proteomic analyses and provided a valuable resource for future comparative studies of venom evolution among venomous hymenopterans.

## 2. Results and Discussion

### 2.1. Transcriptome Analysis

**Figure 1:**
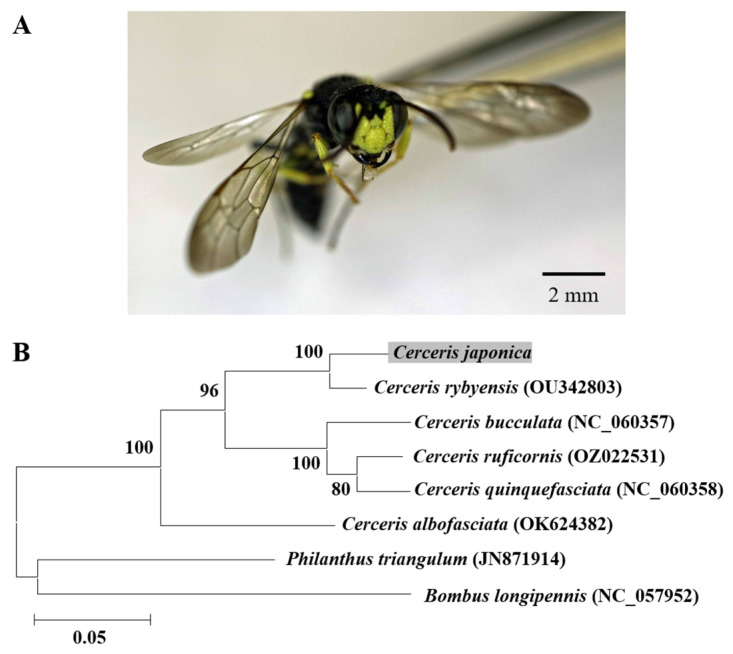
Photograph of *Cerceris japonica* (**A**) and phylogenetic tree using the mitochondrial cytochrome c oxidase subunit I and partial 16S rRNA genes of *C. japonica* and related species (**B**). Since there is limited information on the phylogenetic relationships of *C. japonica* within the genus *Cerceris*, we performed a phylogenetic analysis using mitochondrial DNA sequences from other species in the genus. Numbers above the branches represent bootstrap values (1000 replicates). *C. japonica* is highlighted in gray. Cytochrome c oxidase subunit I and partial 16S rRNA genes were extracted from complete mitochondrial genome sequences. DDBJ/EMBL/GenBank accession numbers are indicated in parentheses for each species, except for *C. japonica*. The accession numbers of *C. japonica* cytochrome c oxidase subunit I and partial 16S rRNA genes are ICXW01000068 and ICXW01000069, respectively.

We examined the phylogenetic placement of *C. japonica* using mitochondrial gene sequences to support species identification and provide an evolutionary context (Figure 1).

Mitochondrial cytochrome c oxidase subunit I and partial 16S rRNA sequences from eight species were aligned and concatenated to construct a phylogenetic tree [7]. Phylogenetic analysis indicated that *C. japonica* is related to *C. rybyensis* (Figure 1). To characterize venom gland gene expression, venom gland mRNA was analyzed using an Illumina HiSeq 6000 platform, yielding 44,548,962 paired-end 100-bp reads. De novo assembly using Trinity generated 68,301 contigs. After clustering highly homologous and redundant sequences using CD-HIT-EST, contigs were annotated using BLAST+ 2.2.30 searches against the UniProtKB and NCBI nucleotide databases to infer their putative functions.

Transcriptome annotation identified 30 toxin-like peptides and proteins, which were classified into nine groups: allergens, chitinases, conotoxin-like peptides, hyaluronidases, phosphatases, phosphodiesterases, phospholipase A_2_s, proteases, and protease inhibitors (Table S1). We have classified toxin-like peptides and proteins according to previous venomics studies based on (i) secretion signals, (ii) homology to known venom proteins, and (iii) proteomic detection in venom [8]. Several toxin-related classes have been previously identified in ant venom [5,6]. Mapping input reads to assembled contigs using Bowtie2 enabled the estimation of transcript abundance. Detailed information on contig identifiers, sequence annotations, and transcript abundance (TPM values) for toxin-like and novel transcripts is provided in Table S1. Among the toxin-like transcripts, venom metalloprotease inhibitor-like protein (comp12158_c0_seq1) showed the highest expression level (TPM, 19471.26). In contrast, linear amphiphilic peptides abundant in some ant venoms were not major transcript components in *C. japonica* venom glands.

Several non-toxin-associated transcripts, including cytochrome P450, elongation factor 1-α, elongation factor 2, ferritin, and vitellogenin, also showed relatively high expression levels in the venom glands. In addition, 11 putative peptides and proteins showed no detectable sequence similarity with known proteins. We designated these proteins and peptides as *Cj* 1–*Cj* 11, although they had no sequence similarities. One of these novel proteins, *Cj* 1 (comp11244_c1_seq1), showed a higher expression level (TPM 21296.75) than that of the venom metalloprotease inhibitor-like protein transcript.

A comparative analysis of previously reported digger-wasp venoms revealed notable differences. Transcripts corresponding to endocuticle structural glycoprotein (comp10723_c0_seq2, TPM 364.24) and icarapin-like protein (comp13545_c0_seq1, TPM 1321.96) were detected at intermediate abundance [2]. Conversely, trehalose-6-phosphate synthase (comp15774_c0_seq1, TPM 68.68), previously reported in other digger wasps [3], showed relatively low abundance. In contrast, transcripts corresponding to FMRFamide-related neuropeptides were not detected in our dataset [1].

### 2.2. Low-Molecular-Weight Components in C. japonica Venom and Venom Sac Extracts

Analysis of low-molecular-weight components suggested the presence of free amino acids (isoleucine/leucine, proline, tyrosine, valine, phenylalanine, and tryptophan) and nucleosides (inosine and guanosine) in the venom and venom sac extracts of *C. japonica*. Histamine and tyramine were detected as sodium adduct ions (Table 1).

Free amino acids are common low-molecular-weight constituents of hymenopteran venoms, although their compositions vary among species [9]. Similarly, nucleosides have been reported in the venom of several solitary wasps. For instance, adenosine has been identified in the venom of *Anoplius samariensis* [10], whereas multiple nucleosides, including adenosine, cytidine, guanosine, thymidine, and uridine, have been reported in *Campsomeriella annulata annulata* [11].

The detection of inosine and guanosine in *C. japonica* further expands the diversity of nucleosides identified in the venom of solitary wasps.

Biogenic amines, such as histamine and tyramine, are widely distributed in hymenopteran venoms [5,9,10,11] and are known to modulate physiological responses through specific receptors. Therefore, the occurrence of these biogenic amines in *C. japonica* venom is consistent with previous observations in other venomous hymenopterans.

Therefore, in addition to peptide and protein toxins, *C. japonica* venom contains diverse low-molecular-weight components that may contribute to venom function.

### 2.3. High-Molecular-Weight Components in C. japonica Venom and Venom Sac Extracts

We compared the liquid chromatography–electrospray ionization–mass spectrometry profiles of crude venom and venom sac extracts under reducing and non-reducing conditions. Under non-reducing conditions, a broad peak at a retention time of 13.88 min showed a deconvoluted neutral mass of 7057.2930 Da, which was in good agreement with the theoretical mass (7057.3070 Da) of the venom metalloprotease inhibitor-like protein.

**Figure 2:**
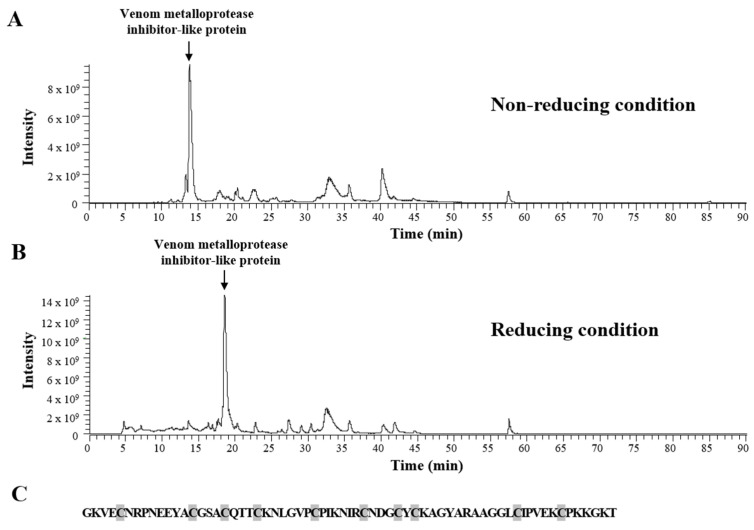
High-molecular-weight components of *Cerceris japonica* venom and venom sac extract are analyzed using LC-ESI-MS. (**A**) Total ion current patterns of *C. japonica* venom and venom sac extract under non-reducing (**A**) and reducing conditions (**B**). Peaks containing venom metalloprotease-like proteins are indicated with arrows. (**C**) Amino acid sequence of venom metalloprotease inhibitor-like protein. The 10 cysteine residues are highlighted in gray.

Following reduction and alkylation with iodoacetamide, the peak shifted to a retention time of 18.53 min and the deconvoluted neutral mass increased to 7637.5731 Da (Figure 2). This increase (580.2935 Da) was consistent with the carbamidomethylation of ten cysteine residues and reduction of five disulfide bonds, yielding the predicted mass of 7637.5865 Da.

Together with the partial peptide sequence matches, characteristic disulfide bonding, and high transcript expression in the venom gland, these results confirmed that these peaks correspond to the venom metalloprotease inhibitor-like protein.

In addition, partial peptide sequences were obtained from ESI–MS/MS analyses performed under reducing and non-reducing conditions with and without tryptic digestion (Table 2). MS/MS spectra were searched using PEAKS 8.5 against components identified from the *C. japonica* venom gland transcriptome.

By integrating the transcriptomic and proteomic datasets, we identified 19 toxin-like peptides and proteins, 14 non-toxin-associated components, and *Cj* 1–11 peptides and proteins. However, proteomic analysis alone did not provide sufficient sequence coverage to determine the presence of signal peptides for most toxin-like proteins and *Cj* 1–11 proteins. Eleven toxin-like proteins, including six metalloendopeptidase-like proteins, one PLA2, two DPPs, one VEGF, and one venom allergen 3 protein, were identified only in the transcriptomic analysis.

Based on its high transcript abundance, *Cj* 1 was expected to be one of the major venom components. However, only peptide fragments derived from *Cj* 1 were detected in the proteomic analysis, possibly because the precursor protein undergoes post-translational proteolytic processing in the venom.

Samples were collected from the dissected venom sacs. These non-toxin-associated components likely represent co-extracted proteins derived from the venom sac.

### 2.4. Biological Activities of Cj 1, Cj 2, and Venom Metalloprotease Inhibitor-like Protein

**Figure 3:**
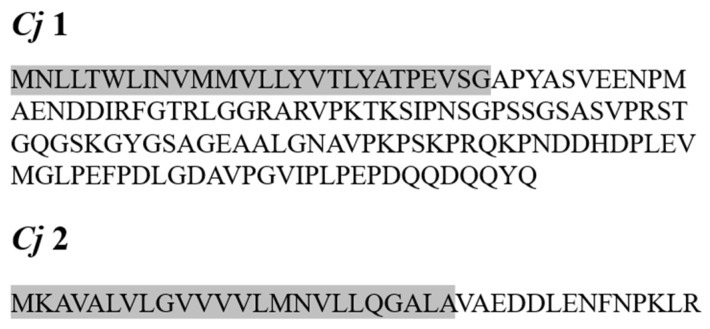
Amino acid sequences of *Cj* 1 and *Cj* 2. Predicted signal sequences are highlighted in gray.

*Cj* 1 was recombinantly expressed in *Escherichia coli* and purified as a 6 × His fusion protein (Figure S1). The venom metalloprotease inhibitor-like protein could not be successfully expressed in *E. coli* and did not form inclusion bodies, possibly because its intrinsic biological activity was detrimental to the host cells. *Cj* 2 was chemically synthesized. The mature forms of *Cj* 1 consisted of 119 amino acid residues with a calculated molecular mass of 12,448.108 Da and *Cj* 2 contained 14 amino acid residues with a molecular mass of 1658.815 Da (Figure 3). Both *Cj* 1 and *Cj* 2 were acidic (calculated pI = 4.77 and 4.16, respectively) and lacked cysteine residues.

**Figure 4:**
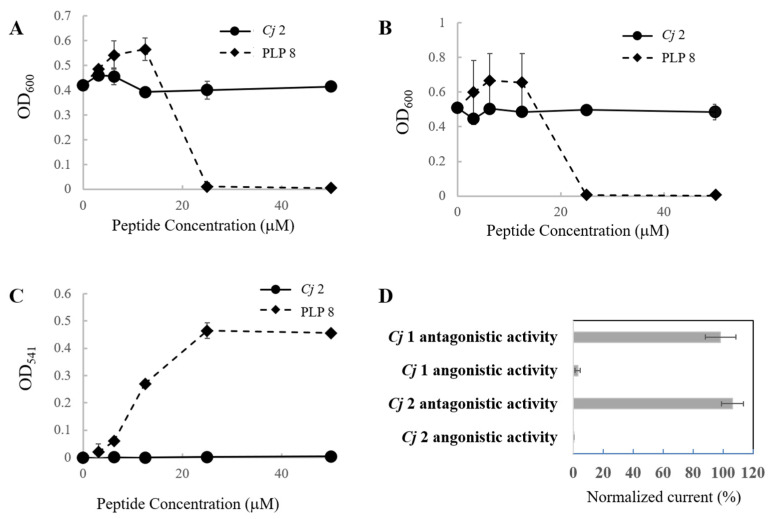
Biological activities of *Cj* 1 and *Cj* 2.Antimicrobial activities against *Escherichia coli* (**A**) and *Staphylococcus aureus* (**B**), and hemolytic activity against rat erythrocytes (**C**) were evaluated using various concentrations of *Cj* 2 and pilosulin-like peptide 8 (positive control). Two independent experiments were performed. Each data point represents the mean of triplicate measurements from a single experiment. Error bars indicate the standard deviation (SD). Effects of *Cj* 1 (50 μM) and *Cj* 2 (1 mM) on nicotinic acetylcholine receptors (nAChRs) (**D**) were evaluated. Responses were normalized to the ACh-induced current recorded from the same α7 nAChR-expressing oocyte. Two independent experiments were performed. Data are presented as the mean ± SEM (*n* = 3 oocytes from a single experiment). Representative data from one of two independent experiments are shown.

*Cj* 1 had no effects on insects at concentrations of 50 μM (*p* = 1.000). A recombinant His-tagged control protein was not included in the functional assays. Although the recombinant *Cj* 1 protein was purified to near homogeneity, additional control proteins may be useful in future studies for further excluding any potential influence of the expression and purification procedures. *Cj* 2 induced paralysis in mealworms in a concentration-dependent manner. The paralytic activities were significantly higher in both the 25 µM and 100 µM treatment groups than in the control group (Fisher’s exact test, *p* = 0.0052 and *p* = 0.0017, respectively) (Table 3). *Cj* 2 significantly increased insecticidal activity at 25 µM (*p* = 0.042). Although a similar tendency was observed at 100 µM, the difference was not statistically significant (*p* = 0.224), possibly because of the limited sample size used in this study. *Cj* 2 showed no hemolytic or antimicrobial activity against *E. coli* or *Staphylococcus aureus* at concentrations up to 50 μM (Figure 4A–C). As some digger wasp toxins target ion channels or ligand-gated receptors, and the philanthotoxins of *p. triangulum* are non-competitive antagonists of nicotinic acetylcholine receptors [12], we examined whether *Cj* 1 and *Cj* 2 modulate nAChRs. *Cj* 1 and *Cj* 2 showed no effect on rat nAChR α7 at 50 µM and 1 mM, respectively (Figure 4D and Figure S2). We could not detect intact *Cj* 1 in the LC–MS analysis without trypsin treatment. *Cj* 1 is likely processed after translation, and the processed form may possess biological activity. Further studies are required to prove this hypothesis.

**Table 3:** Paralytic and insecticidal effects of *Cj* 1 and *Cj* 2.

Treatment	Paralyzed (Alive) ^a,b^	Dead ^a,c^	Unaffected ^a^
Insect ringer solution	1	0	14
*Cj* 1 (50 μM)	0	0	15
*Cj* 2 (25 μM)	9	5	6
*Cj* 2 (100 μM)	10	3	5

^a^ Number of insects; ^b^ number of paralyzed insects 1 h after injection; ^c^ number of dead insects 16 h after injection.

Both venom metalloprotease inhibitor-like protein and *Cj* 1 are major components of the venom of this species.

Venom metalloprotease inhibitor-like proteins in frogs and scorpions have been reported to inhibit trypsin, chymotrypsin, and elastase [13,14]. Moreover, CvT-TIL from a parasitoid wasp, which possesses a trypsin inhibitor-like (TIL) cysteine-rich domain similar to that found in venom metalloprotease inhibitor-like proteins, suppresses the immune response by inhibiting hemolymph prophenoloxidase activation in moth larvae [15]. By suppressing host immune responses, parasitoid wasps may preserve their hosts for extended periods. Taken together with the absence of linear cationic α-helical peptides, which are common components of ant venoms [5,6], these observations raise the possibility that the venom composition of *C. japonica* shares greater similarity with that of parasitoid wasps than with that of ants.

Unlike ants and honeybees, which generally do not need to keep their prey alive for extended periods, the digger wasp *C. japonica* needs to keep its prey alive for several weeks to provide fresh food for its larvae. Therefore, the composition of its venom may have evolved in association with its hunting behavior rather than solely reflecting its phylogenetic relationships.

However, because *C. japonica* is a prey-specialized species and comparative venom datasets from other *Cerceris* species remain limited, this hypothesis should be interpreted with caution. Our dataset provides a valuable resource for future comparative analyses of venom evolution among predatory wasps, parasitoid wasps, bees, and ants.

## 3. Conclusions

In this study, we characterized the venom of *C. japonica* using transcriptomic and proteomic analyses. We identified diverse low- and high-molecular-weight components, including toxin-like peptides and proteins, in addition to 11 novel components (*Cj* 1–11) with no detectable similarity to known peptides or proteins. The venom metalloprotease inhibitor-like protein and *Cj* 1 showed relatively high transcript abundance. The synthetic peptide *Cj* 2 exhibited paralytic activity against mealworms. These findings expand our current knowledge of digger wasp venoms and provide a basis for future studies to elucidate the biological roles of the major venom components.

## 4. Materials and Methods

### 4.1. Study Species

*C. japonica* specimens were collected from Tokyo Metropolitan Mizumoto Park, Tokyo, Japan, during their nesting season. One insect (24 September 2018) was used for transcriptome analysis and three insects (8 August 2019) were used for proteome analysis. These specimens were identified as *C. japonica* based on their morphological characteristics, including their distinctive black abdominal markings and yellow banding patterns. Species identity was further supported by a mitochondrial gene-based phylogenetic analysis. A phylogenetic tree was constructed using the maximum-likelihood method with the Tamura–Nei model in MEGA version 12 [16].

### 4.2. Transcriptome Analysis

Total RNA was isolated from the venom glands and sacs using TRIzol reagent (Thermo Fisher Scientific, Waltham, MA, USA). Paired-end cDNA libraries were constructed using a TruSeq Stranded mRNA kit (Illumina, San Diego, CA, USA), and sequencing was performed using an Illumina NovaSeq 6000 (Illumina) by Macrogen Japan Corp. (Kyoto, Japan). After the adapter and low-quality sequences were eliminated using Trimmomatic 0.36 [17], the transcriptome data were assembled using Trinity (v.2.1.1) [18]. All input reads were mapped to the contigs and counted using Bowtie 2 to estimate the relative expression levels of the venom gland transcripts.

### 4.3. LC–MS Analysis for Low-Molecular-Weight Components

Three *C. japonica* venom sacs were dissected by grasping the sting with forceps and subsequently extracted in 50% acetonitrile containing 0.1% (*v*/*v*) trifluoroacetic acid (50 µL) for 2 h at 4 °C. The resulting extract was filtered through a 0.45 µm membrane filter and then diluted 20-fold with the extraction solvent for LC–MS analysis. Liquid chromatography was performed under the following conditions: solvent A, 0.1% (*v*/*v*) aqueous formic acid; solvent B, 0.1% (*v*/*v*) formic acid in acetonitrile; 5–65% linear gradient of solvent B in solvent A at a flow rate of 200 µL/min; column, Capcell Pak C18 UG 120 (1.5 × 150 mm, Shiseido, Tokyo, Japan); column temperature, 25 °C. The molecular masses of the venom peptides were determined using an LTQ Orbitrap XL-ETD (Thermo Fisher Scientific). Mass spectrometric analysis was carried out under the following conditions: ionization, electrospray in positive mode; ion spray voltage, 4.6 kV; capillary temperature, 350 °C; capillary and tube lens voltages, 19 and 35 V, respectively; detector, an Orbitrap at a resolution of 60,000 at *m*/*z* 400. The MS scan range was 100–1500 *m*/*z*. The instrument was calibrated by polytyrosine, and mass accuracy was typically 1–3 ppm after calibration.

### 4.4. LC–MS Analysis for Peptide and Protein Components

Following filtration, the venom sac extract was diluted 10-fold with 50 mM ammonium bicarbonate (pH 8.0) to improve chromatographic separation and reduce peak tailing during high-performance liquid chromatography (HPLC). The diluted extract was subsequently reduced with dithiothreitol (10 mM; Thermo Fisher Scientific), alkylated with iodoacetamide (20 mM; Thermo Fisher Scientific), or digested with trypsin (30 µg/mL; Promega, Madison, WI, USA) overnight at 37 °C. Peptides were separated on a Zaplous α pep C18 analytical column (3 µm, 120 Å, 1.5 × 150 mm, AMR, Tokyo, Japan) with an L-Trap column (5 µm, 0.3 × 5 mm, AMR) at a flow rate of 500 µL/min using two mobile phases, 0.1% (*v*/*v*) aqueous formic acid (solvent A) and 0.1% (*v*/*v*) formic acid in acetonitrile (solvent B). The gradient program was as follows: 0–60 min, 5–65% solvent B; 60–70 min, 65–95% solvent B; and 70–80 min, 95% solvent B. The molecular masses of the wasp peptides were determined using a Q Exactive (Thermo Fisher Scientific). Mass spectrometric analysis was performed under the following conditions: ionization, nano-electrospray (CaptiveSpray Ionization: CSI) in positive mode; ion spray voltage, 1.4 kV; capillary temperature, 250 °C; S-lens level, 50; detector, an Orbitrap at a resolution of 70,000 from 350–2000 *m*/*z*. The instrument was calibrated using LTQ Velos ESI Positive Ion Calibration Solution (Thermo Fisher Scientific), and the mass accuracy was usually 1–3 ppm after measurement. Raw mass data were processed using Xcalibur (Thermo Fisher Scientific). Peptide sequences were assigned from the MS/MS spectra using PEAKS 8.5 (Bioinformatics Solutions, Waterloo, ON, Canada; parent mass error tolerance 10.0 ppm, fragment mass error tolerance 0.02 Da, and score threshold 15.0) and subsequently verified manually.

We selected 67 amino acid sequences derived from major transcripts in the venom gland transcriptome analysis and 116 amino acid sequences of common external contaminants from the Global Proteome Machine Organization. The selected sequences were compiled into a FASTA format file and incorporated into the PEAKS 8.5 software as a reference database.

### 4.5. Preparation of Recombinant Cj 1 Protein and Synthesis of Cj 2 Peptides

The *Cj* 1 cDNA fragment was amplified using PCR with a forward primer *Cj* 1S [5′-CCGGATCCCTGGAAGTTCTGTTCCAGGGGCCCGCACCATACGCTTCAGTC-3′: *Bam*HI site is underlined; the PreScission (Cytiva, Marlborough, MA, USA) recognition amino acid sequence (Leu-Glu-Val-Leu-Phe-Gln-Gly-Pro) is double-underlined] and a reverse primer *Cj* 1AS (5′-CCCTGCAGTTACTGGTACTGTTGATC-3′: *Pst*I site is underlined). After the amplified DNA was digested with *Bam*HI and *Pst*I, the products were subcloned into the pCold II expression vector (Takara, Otsu, Japan). The recombinant protein expression and purification were performed as previously described [19].

The *Cj* 2 peptide was prepared via Fmoc chemistry using GenScript (Nanjing, China). Peptides were purified using reverse-phase HPLC with a preparative C18 column. The purity and molecular weight of the final peptides were verified using HPLC and MS, respectively.

### 4.6. Western Blotting

To verify the identity of the purified His-tagged recombinant *Cj* 1 protein, Western blotting was performed using a Penta-His antibody (Qiagen, Hilden, Germany) as the primary antibody and an HRP-conjugated anti-mouse IgG antibody (Clarivate, London, UK) as the secondary antibody. Chemiluminescent signals were detected using ImmunoStar LD (FUJIFILM Wako Pure Chemical Corporation, Osaka, Japan) and visualized with a C-DiGit blot scanner (LI-COR Biotechnology, Lincoln, NE, USA).

### 4.7. Biological Assays

Antimicrobial, hemolytic, and electrophysiological assays were performed as previously described [5,19]. Insecticidal activity was assessed as follows: sixty mealworms (*Tenebrio molitor*, 80–100 mg) were randomly assigned to four groups (*n* = 15 per group): control, *Cj* 1 (50 µM), and *Cj* 2 (25 µM and 100 µM). The mealworms were anesthetized on ice, and the test solutions were injected into the hemocoel using a 0.5 mL insulin syringe fitted with a 30-gauge needle. Ten microliters of insect Ringer solution (5 mM HEPES (pH 7.4), 35 mM NaCl, 36 mM KCl, 12 mM CaCl_2_, 16 mM MgCl_2_, and 274 mM glucose), *Cj* 1 solution, or *Cj* 2 solution were injected into the hemocoel of mealworms in each group. The experiment was independently performed twice. Paralytic and insecticidal activities were compared using a two-tailed Fisher’s exact test. Paralysis and mortality were recorded after 16 h.

## Figures and Tables

**Table 1:** Low-molecular-mass components of *Cerceris japonica* venom and venom sac extract as analyzed using LC–ESI–MS.

Compound	RT (min)	Formula	Observed *m*/*z*	Calculated *m*/*z*
Proline	4.18	C_5_H_9_NO_2_	116.0710	116.0712
Valine	4.58	C_5_H_11_NO_2_	118.0867	118.0868
Tyrosine	5.48	C_9_H_11_NO_3_	182.0817	182.0817
Isoleucine/ Leucine	5.74	C_6_H_13_NO_2_	132.1022	132.1025
Phenylalanine	6.98	C_9_H_11_NO_2_	166.0866	166.0868
Tryptophan	10.4	C_11_H_12_N_2_O_2_	205.0974	205.0977
Inosine	5.23	C_10_H_12_N_4_O_5_	269.0880	269.0886
Guanosine	5.34	C_10_H_13_N_5_O_5_	284.0990	284.0995
Histamine	10.48	C_5_H_9_N_3_	134.0604 *	134.0694 *
Tyramine	10.43	C_8_H_11_NO	160.0759 *	160.0738 *

* *m*/*z* of [M + Na]+. LC–ESI–MS: liquid chromatography–electrospray ionization–mass spectrometry.

**Table 2:** LC analyzes high-molecular-mass components of *C. japonica* venom and venom sac extract–ESI–MS.

	Peptide/Protein	Accession Number	Coverage (%) ^a^			
			TR ^b^−, DTT ^b^−	TR−, DTT+	TR+, DTT−	TR+, DTT+
Toxin-like peptides and proteins	Chitinase	ICXW01000001	6	10	78	78
	Hyaluronidase	ICXW01000002	0	2	65	87
	Membrane metalloendopeptidase-like	ICXW01000003	5	0	50	33
	Neprilysin-like	ICXW01000010	5	0	35	10
	Trypsin-like protease	ICXW01000011	100	47	46	79
	PLA_2_-1	ICXW01000012	10	0	13	8
	PLA_2_-2	ICXW01000013	3	10	4	3
	PLA_2_-3	ICXW01000014	11	13	27	19
	Sphingomyelin phosphodiesterase	ICXW01000016	6	0	2	0
	Venom acid phosphatase-1	ICXW01000017	26	0	0	0
	Venom acid phosphatase-2	ICXW01000018	7	14	0	0
	Venom acid phosphatase-3	ICXW01000019	10	0	3	0
	Venom acid phosphatase-4	ICXW01000020	19	16	39	15
	Actitoxin-like peptide (Kunitz-type protease inhibitor)	ICXW01000023	0	86	14	88
	Venom metalloproteinase inhibitor-like protein	ICXW01000024	9	98	28	65
	Whey acidic protein (protease inhibitor)	ICXW01000025	0	0	5	67
	Conotoxin L1	ICXW01000026	0	49	0	12
	Conotoxin S1	ICXW01000027	0	0	0	39
	Icarapin-like protein	ICXW01000028	0	0	30	0
Non-toxin-associated peptides and proteins *	Calmodulin	ICXW01000031	0	0	66	85
	Carboxylesterase-1	ICXW01000032	15	6	1	0
	Carboxylesterase-2	ICXW01000033	0	2	2	0
	Glyceraldehyde 3-phosphate dehydrogenase 2	ICXW01000035	12	0	65	91
	Pancreatic triacylglycerol lipase-1	ICXW01000036	2	15	5	0
	Pancreatic triacylglycerol lipase-2	ICXW01000037	12	0	0	0
	Heat shock 70 kDa protein	ICXW01000046	0	4	71	71
	Histone	ICXW01000047	54	21	61	54
	Myosin heavy chain	ICXW01000053	8	2	57	49
	RNA polymerase	ICXW01000038	0	0	9	22
	Eukaryotic translation initiation factor 5B	ICXW01000049	0	0	5	0
	Tropomyosin 1	ICXW01000054	25	0	77	91
	Troponin C	ICXW01000055	0	0	31	9
	Vitellogenin	ICXW01000056	9	3	65	60
Novel peptides and proteins (no homology)	*Cj* 1	ICXW01000057	100	99	81	77
	*Cj* 2	ICXW01000058	89	50	32	34
	*Cj* 3	ICXW01000059	99	92	63	73
	*Cj* 4	ICXW01000060	6	68	74	86
	*Cj* 5	ICXW01000061	82	45	74	68
	*Cj* 6	ICXW01000062	73	56	70	70
	*Cj* 7	ICXW01000063	24	16	55	44
	*Cj* 8	ICXW01000064	31	65	69	87
	*Cj* 9	ICXW01000065	5	70	57	64
	*Cj* 10	ICXW01000066	0	42	38	97
	*Cj* 11	ICXW01000067	0	28	71	80

^a^ The coverages were calculated by combining the peptide fragments with the same amino acid sequences and same molecular masses using PEAKS 8.5; ^b^ TR and DTT indicate MS/MS data with or without trypsin digestion and DTT treatment, respectively. * Proteins may have been derived from the venom sac.

## Data Availability

The original contributions presented in this study are included in the article/Supplementary Materials. Further inquiries can be directed to the corresponding author.

## Supplementary Materials

The following supplementary data are available online at: https://www.mdpi.com/article/10.3390/toxins18070307/s1, Figure S1. Purification of recombinant His-tagged *Cj* 1 protein; Figure S2. Effects of *Cj* 1 and *Cj* 2 on neuronal α7 nAChRs expressed in oocytes; Table S1. Major 67 components of *C. japonica* identified by venom gland transcriptome analysis.

## Abbreviations

The following abbreviations are used in this manuscript:

COI	cytochrome c oxidase subunit I
LC–ESI–MS	liquid chromatography–electrospray ionization–mass spectrometry
HPLC	high-performance liquid chromatography
