# Supplementary material for: Integrated Venom Gland Transcriptomic and Venom Proteomic Analyses of the Digger Wasp Cerceris japonica
Source: Toxins (Basel). 2026 Jul 15;18(7):307. doi: 10.3390/toxins18070307 (PMC13418622; doi:10.3390/toxins18070307)
Supplement: Supplementary file 1 [file toxins-18-00307-s001.zip › toxins-4358345-supplementary.pdf]

Supplementary Materials: Integrated venom gland transcriptomic and venom proteomic analyses of the digger wasp *Cerceris japonica*

Kohei Kazuma, Naoki Tani, Katsuhiro Konno, Migaku Kawaguchi, and Hidetoshi Inagaki

Table S1. Major 67 components of *C. japonica* identified by venom gland transcriptome analysis.

Peptide/Protein	Contig ID	Accession Number*	TPM	Theoretical mass[a]
Chitinase	comp13511_c0_seq1	ICXW01000001	2792.93	48067.531
Hyaluronidase	comp10734_c0_seq1	ICXW01000002	877.34	26290.523
Membrane metallo endopeptidase-like	comp13310_c0_seq1	ICXW01000003	402.14	26066.328
Metallo-endopeptidase-like-1	comp4740_c0_seq1	ICXW01000004	349.99	84047.17
Metallo-endopeptidase-like-2	comp17201_c0_seq1	ICXW01000005	195.33	80426.801
Metallo-endopeptidase-like-3	comp16302_c1_seq7	ICXW01000006	62.25	56458.874
Metallo-endopeptidase-like-4	comp16481_c2_seq2	ICXW01000007	75.93	60637.717
Metallo-endopeptidase-like-5	comp14968_c0_seq1	ICXW01000008	84.44	83951.936
Metallo-endopeptidase-like-6	comp16386_c0_seq7	ICXW01000009	23.19	45175.563
Neprilysin-like	comp17545_c0_seq1	ICXW01000010	69.94	26285.026
Trypsin-like protease	comp16935_c0_seq1	ICXW01000011	6160.77	23337.346
PLA_2-1	comp13428_c0_seq4	ICXW01000012	2.93	25261.648
PLA_2-2	comp15137_c1_seq1	ICXW01000013	23.66	24729.969
PLA_2-3	comp16489_c0_seq55	ICXW01000014	2.41	24899.187
PLA_2-4	comp14761_c0_seq4	ICXW01000015	29.77	25358.494
Sphingomyelin phosphodiesterase	comp18585_c0_seq1	ICXW01000016	26.17	26199.33
Venom acid phosphatase-1	comp5127_c0_seq1	ICXW01000017	33.84	25980.963
Venom acid phosphatase-2	comp15245_c1_seq21	ICXW01000018	17.69	26120.306
Venom acid phosphatase-3	comp9660_c0_seq1	ICXW01000019	43.38	25888.449
Venom acid phosphatase-4	comp6557_c0_seq1	ICXW01000020	124.43	25814.62
Venom dipeptidyl peptidase 4-1	comp13720_c0_seq1	ICXW01000021	68.42	87295.768
Venom dipeptidyl peptidase 4-2	comp3276_c0_seq1	ICXW01000022	11.07	90346.443
Actitoxin-like peptide (Kunitz type protease inhibitor)	comp16882_c0_seq2	ICXW01000023	648.77	9117.41
Venom metalloproteinase inhibitor-like protein	comp12158_c0_seq1	ICXW01000024	19471.26	9316.6
Whey acidic protein	comp12018_c0_seq1	ICXW01000025	285.81	12674.008
Conotoxin L1	comp16963_c0_seq1	ICXW01000026	1300.99	8629.241
Conotoxin S1	comp21878_c0_seq1	ICXW01000027	6.7	7029.28
Icarapin-like protein	comp13545_c0_seq1	ICXW01000028	1321.96	23935.615
Venom allergen 3	comp4679_c0_seq1	ICXW01000029	8.32	24974.448
VEGF-like	comp14995_c0_seq1	ICXW01000030	25.37	92030.7
Calmodulin	comp5452_c0_seq1	ICXW01000031	326.05	16799.813
Carboxylesterase-1	comp16828_c1_seq4	ICXW01000032	5.09	24674.44
Carboxylesterase -2	comp11005_c0_seq1	ICXW01000033	436.94	24807.661

Cytochrome P450	comp4697_c0_seq1	ICXW01000034	20777.99	62930.702
Glyceraldehyde 3 phosphate dehydrogenase 2	comp16990_c0_seq1	ICXW01000035	1809.65	23814.189
Pancreatic triacylglycerol lipase-1	comp14734_c0_seq1	ICXW01000036	19.05	24627.475
Pancreatic triacylglycerol lipase-2	comp12407_c0_seq1	ICXW01000037	150.33	25081.365
RNA polymerase	comp16872_c4_seq11	ICXW01000038	8.43	26904.187
Trehalose-6-phosphate synthase	comp15774_c0_seq1	ICXW01000039	68.68	90588.652
Elongation factor 1-alpha	comp16939_c0_seq1	ICXW01000040	3847.48	50284.354
Ferritin	comp4700_c0_seq1	ICXW01000041	2633.29	24075.316
FKBP-1	comp12311_c0_seq1	ICXW01000042	213.25	21951.237[b]
FKBP-2	comp11751_c0_seq1	ICXW01000043	51.17	25638.663
FKBP-3	comp7401_c0_seq1	ICXW01000044	26.16	42526.052
FKBP-4	comp15516_c0_seq3	ICXW01000045	22.4	51407.29
Heat shock 70 kDa protein	comp16605_c0_seq3	ICXW01000046	87.06	71622.998
Histone	comp17495_c0_seq1	ICXW01000047	41.43	15259.418[b]
Elongation factor 2	comp11415_c0_seq1	ICXW01000048	2200.57	94488.964
Eukaryotic translation initiation factor 5B	comp15151_c0_seq1	ICXW01000049	14.93	25008.502[b]
Defensin	comp16912_c0_seq36	ICXW01000050	75.7	10972.252
Defensin	comp16827_c0_seq7	ICXW01000051	60.87	10676.279
Endocuticle structural glycoprotein	comp10723_c0_seq2	ICXW01000052	364.24	17352.37
Myosin heavy chain	comp15243_c0_seq4	ICXW01000053	255.28	224785.858
Tropomyosin 1	comp17197_c0_seq1	ICXW01000054	187.26	17399.428
Troponin C	comp4738_c0_seq1	ICXW01000055	361.83	25192.641[b]
Vitellogenin	comp12210_c1_seq1	ICXW01000056	10258.94	25059.656
Cj 1	comp11244_c1_seq1	ICXW01000057	21296.75	15506.713
Cj 2	comp16938_c0_seq1	ICXW01000058	1089.02	4061.268
Cj 3	comp16900_c2_seq1	ICXW01000059	315.86	10874.604
Cj 4	comp12569_c0_seq2	ICXW01000060	1429.76	24895.447
Cj 5	comp15263_c0_seq5	ICXW01000061	144.63	22389.62
Cj 6	comp4704_c0_seq1	ICXW01000062	4173.25	10120.992
Cj 7	comp16916_c0_seq6	ICXW01000063	151.36	25564.707
Cj 8	comp12569_c0_seq1	ICXW01000064	1833.05	24715.714
Cj 9	comp16944_c0_seq1	ICXW01000065	3035.33	24687.146
Cj 10	comp13460_c1_seq1	ICXW01000066	1453.22	24477.114
Cj 11	comp16421_c0_seq1	ICXW01000067	465.22	24947.848

*DDBJ/EMBL/GenBank accession numbers for the corresponding nucleotide sequences.

[a] Most theoretical molecular masses were calculated from full-length protein sequences.

[b] Some molecular masses were calculated from partial protein sequences because the 5′ or 3′ ends of the corresponding transcripts were truncated.

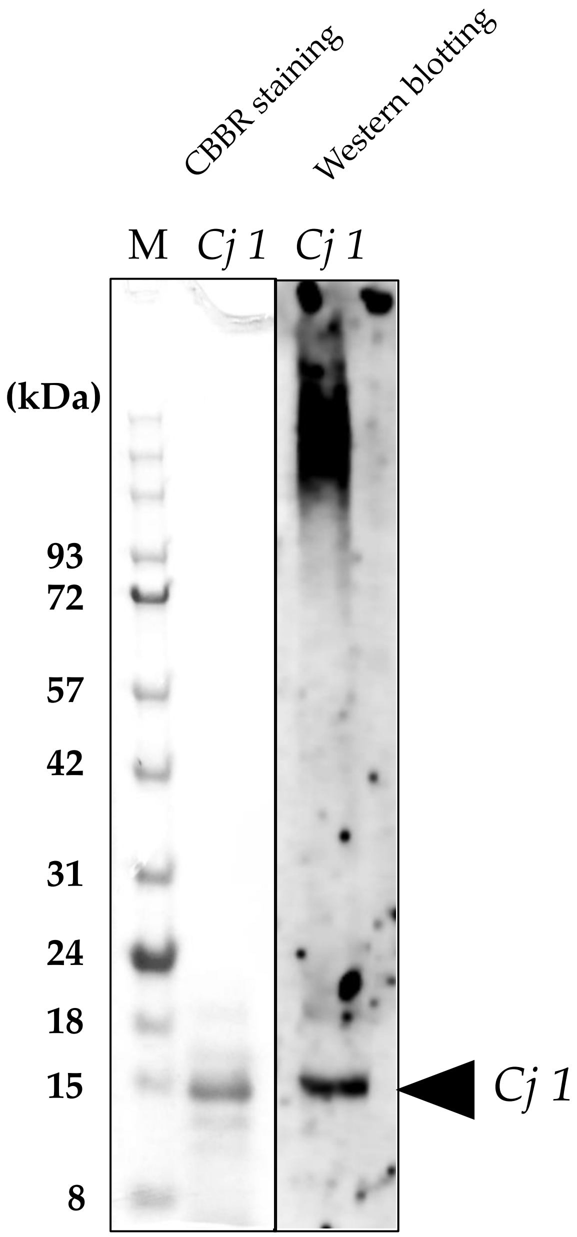

Figure S1. Purification of recombinant His-tagged *Cj* 1 protein. Recombinant His-tagged *Cj* 1 protein was purified using TALON affinity chromatography. After buffer exchange using a PD-10 desalting column, the purified protein was analyzed on a 4–12% NuPAGE gel and stained with Coomassie Brilliant Blue R-250 (A). His-tagged *Cj* 1 protein was detected by Western blotting using a Penta-His antibody (B). M, molecular mass marker; *Cj* 1, purified recombinant *Cj* 1 protein. Because the recombinant protein contains vector-derived residues, including the His tag, and is acidic, it migrated with an apparent molecular mass higher than the predicted molecular mass of mature *Cj* 1.

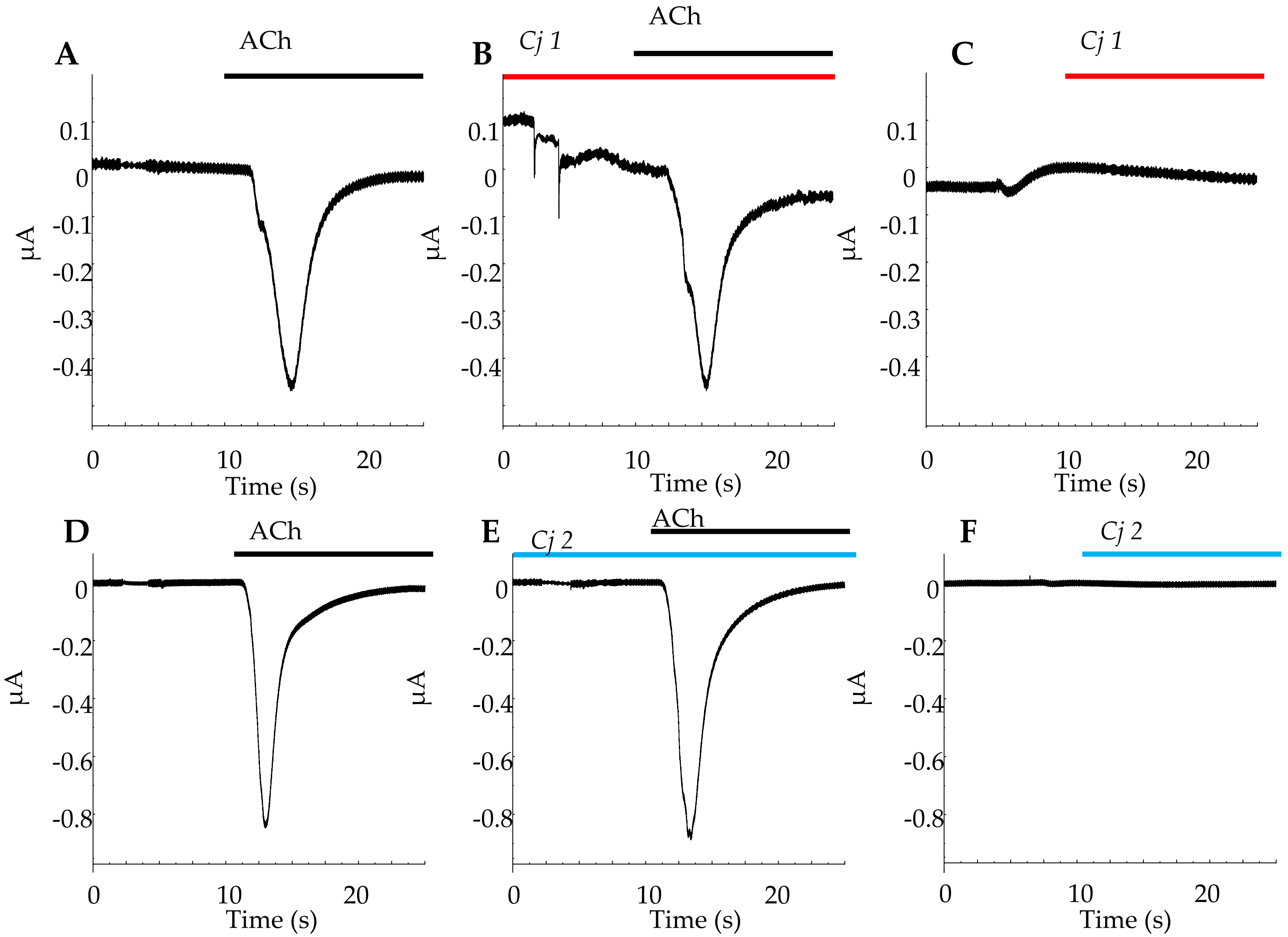

Figure S2. Effects of *Cj* 1 and *Cj* 2 on neuronal α7 nAChRs expressed in oocytes.

Acetylcholine-evoked currents mediated by neuronal α7 nAChRs were recorded in the absence (A, D) or presence of *Cj* 1 (B) or *Cj* 2 (E). Responses of neuronal α7 nAChRs to *Cj* 1 and *Cj* 2 alone were examined (C, F). Black, red, and light blue bars indicate the application of acetylcholine (10 μM), *Cj* 1 (50 μM), and *Cj* 2 (1 mM), respectively. *Cj* 1 and *Cj* 2 showed no effect on rat nAChR α7 at 50 mM and 1 mM, respectively (B, C, E, F).
